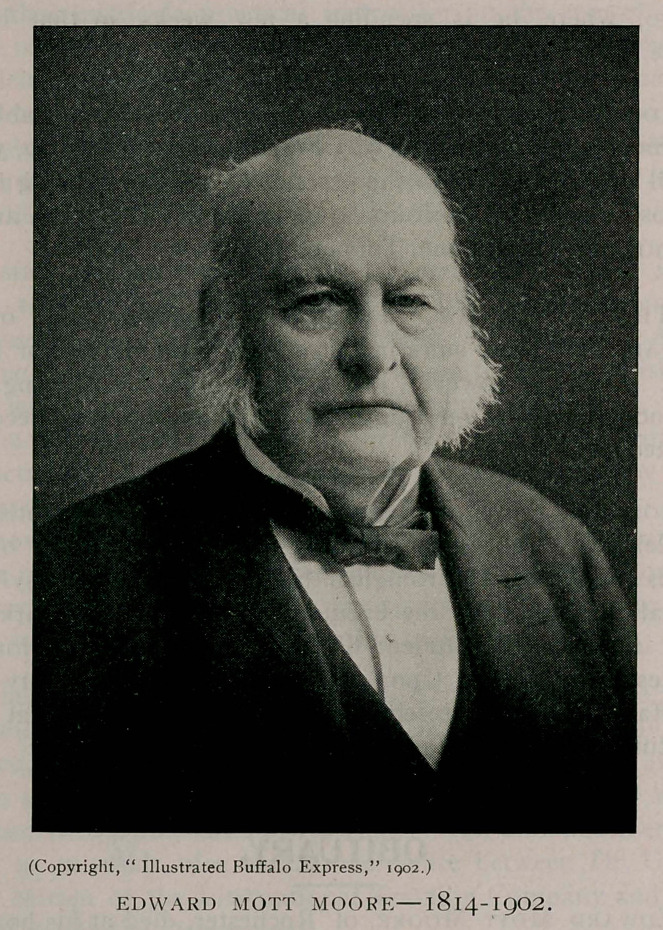# Dr. Edward Mott Moore

**Published:** 1902-04

**Authors:** 


### OBITUARY.

Dr. Edward Mott Moore, of Rochester, died at his home in that city March 3, 1902, aged 88 years. He was born in Rahway, N. J., in 1814, of French Huguenot and English parentage. His family moved to this city in 1830, and he was graduated from the medical school of the University of Pennsylvania in 1838 and began the practice of medicine in Rochester.

Dr. Moore then began a medical and surgical career that was destined to make him famous throughout the English speaking world. In 1843, he was elected to the chair of surgery in the medical college at Woodstock, Vt., and in 1858 was chosen professor of surgical pathology in the medical department of the University of Buffalo. In 1860, upon the removal of Professor Frank Hastings Hamilton to New York, Dr. Moore succeeded to the chair of principles and practice of surgery, in which he continued to teach for the next twenty-five years. In 1880 he

(Copyright, "Illustrated Buffalo Express," 1902.)

EDWARD MOTT MOORE—1814-1902.

was appointed president of the state board of health in accordance with the law then passed creating that body. He retired from the chair of surgery at Buffalo in 1883, but continued his service at the state board of health until some years later.

Dr. Moore is best known to the professional world as a surgeon, and during his extensive career as a teacher and practitioner of the art he contributed many important methods of dealing with injuries, especially as related to fractures of the long bones. Conspicuously among the number may be mentioned his unique appliance for the treatment of Colles's fracture of the radius and his dressings for the fracture of the clavicle. He also was a contributor to medical literature and his monographs will stand out with conspicuous prominence as having added to the sum of professional knowledge.

He was president of the Medical Society of the State of New York in 1874 and of the American Medical Association in 1888, and of the American Surgical Association in 1883. He was also for many years president of the board of trustees of Rochester University.

In Buffalo, Dr. Moore was well known and during his twenty-five years of teaching at the university had endeared to himself many pupils, now scattered all over the world, who will ever revere his memory. Appropriate action was taken by the faculty of Buffalo University and the Buffalo Academy of Medicine.

## Figures and Tables

**Figure f1:**